# Myeloid Sarcoma as a Presentation of Acute Myeloid Leukemia and Blastic Phase in the Course of Chronic Myeloid Leukemia: A Case Report and Literature Review

**DOI:** 10.3390/jcm12144845

**Published:** 2023-07-23

**Authors:** Ugo Giordano, Mateusz Sawicki, Justyna Pilch, Jakub Mizera, Artur Borkowski, Krzysztof Zduniak, Jarosław Dybko

**Affiliations:** 1 Department of Nephrology and Transplant Medicine, University Clinical Hospital in Wroclaw, 50-556 Wrocław, Poland; 2 Faculty of Medicine, Wroclaw Medical University, 50-367 Wroclaw, Poland; justyna.pilch@student.umw.edu.pl (J.P.); jakub.mizera@student.umw.edu.pl (J.M.); 3 Lower Silesian Centre for Oncology, Pulmonology and Hematology in Wrocław, 53-439 Wroclaw, Poland; sawicmat@gmail.com (M.S.); kzdun91@gmail.com (K.Z.); dybko@post.pl (J.D.); 4 Department of Nuclear Medicine and Endocrine Oncology, M. Sklodowska-Curie National Research Institute of Oncology Gliwice Branch, 44-102 Gliwice, Poland; artur.borkowski.md@gmail.com

**Keywords:** myeloid sarcoma, acute myeloid leukemia, chronic myeloid leukemia, chloroma, granulocytic sarcoma, case report

## Abstract

Background: Myeloid sarcoma (MS), also known as granulocytic sarcoma or chloroma, is a rare disease entity characterized by the emergence of an extramedullary tumor, which may be antecedent, coexisting, or manifest secondarily to an ongoing malignancy of lymphoid origin. Owing to its low prevalence, scientific reports addressing this matter comprise mainly retrospective studies with a limited number of participants, rather low-quality research, and only few case reports. Despite MS’s rarity, the need for enhancing their diagnostic tools and refinement of their therapeutic regimens is broadly recognized among physicians. Case summary: In this case series, we present the clinical histories of two patients diagnosed with MS. The former (Case 1) exhibited MS of the sternum alongside chronic myeloid leukemia (CML), while in case of the latter (Case 2) MS presented as the initial manifestation of a current acute myeloid leukemia (AML). Treatment for both patients included chemotherapy (CHTH) and radiation (RT); however, patient 1 with CML died due to cardiorespiratory insufficiency secondary to an infection, while patient 2 is in clinical remission (CR) for 16 months since their MS diagnosis. Furthermore, a comprehensive analysis of previously reported cases was conducted which incorporated MS in patients with AML and CML. Conclusion: The objective of this report was to emphasize the heterogeneity among the clinical manifestations of MS, to underline the relevance of the histopathological and molecular diagnostic tools in opting for the appropriate therapy, and that, in spite of it occurring rather uncommonly, physicians should think of MS in the presence of pathological masses in patients under risk of hematological malignancies.

## 1. Introduction

According to the World Health Organization (WHO) classification of hematolymphoid tumors, MS presents as a distinctive tissue-based manifestation of AML, transformed myelodysplastic syndrome (MDS), myeloproliferative neoplasms (MPN) or MDS/myeloproliferative neoplasms (MPN) [1]. The majority of individuals with MPN are typically identified in the chronic phase (CP), during which there is a possibility of progressing onto the blast phase (BP). The BP is characterized by the presence of additional cytogenetic and/or molecular abnormalities. In view of the *5th edition of the WHO Classification of Hematolymphoid Tumors*, MS can be interpreted as a manifestation of the BP in CML, as one of the diagnostic criteria of the BP is the presence of an abnormal proliferation of blasts outside the bone marrow [1]. Similarly, according to the latest *International Consensus Classification of Myeloid Neoplasms and Acute Leukemias*, one of the diagnostic criteria of the BP in CML is MS, which has been defined as an extramedullary blast proliferation. There have also been reports in which MS ensued without bone marrow (BM) involvement, in which case it is termed as isolated myeloid sarcoma [2]. Cases of newly occurring myeloid sarcoma should undergo a thorough investigation that includes cytogenetic and molecular analyses to permit accurate classification and effective treatment planning. Approximately 70% of patients exhibit concordant molecular alterations in both myeloid sarcoma and concurrent bone marrow disease, implying a potential origin from a shared hematopoietic stem cell or precursor [3,4]. In some individuals with a morphologically normal BM, relevant gene mutations can be still detected, indicating the presence of a low-level clonal myeloid disease or clonal hematopoiesis within the bone marrow [3,5]. It has been found that there is a prevalence of males over females in patients affected by MS, of whom approximately 9% are at risk of developing AML or CML [6]. Due to its rarity and lack of prospective and randomized controlled studies, data concerning the prognosis is conflicting, and there is no consensus on the treatment. In addition, MS is a source of significant diagnostic burden, which results in frequent misdiagnosis with various malignancies, such as Non-Hodgkin’s Lymphoma (NHL), small round cell tumors, or thymoma, which significantly delays treatment institution [7]. MS can be interpreted in various manners depending on its time of manifestation in regard to that of AML or CML. If it presents primarily or concomitantly, MS may be perceived as a manifestation of an ongoing hematological malignancy outside of the BM. However, if it is secondary, it could be seen a sign of disease progression and development of resistance to therapy. As for the applied regimen, it is worth mentioning in that it could undergo modifications over time due to the presence of mutations that may appear as the disease progresses. As a result, the initially administered medications become ineffective. Through an in-depth analysis of our cases along with previous studies concerning MS in patients with AML or CML, we sought to provide further insights into both the diagnostic and therapeutic approaches for the management of patients afflicted by MS, and emphasize the fundamental role played by molecular testing in opting for the adequate, most efficacious therapy. Moreover, we explored whether MS occurs rather primarily or secondarily to myeloid malignancies, the most frequently affected sites, and how its manifestation may influence our proceedings.

## 2. Materials and Methods

All patients provided informed consent for both the procedures itself and the publication of the resulting data. To gather the relevant information, an extensive literature search was conducted, encompassing publications up until June 2023. Various combinations of keywords, such as myeloid sarcoma, AML, CML, chloroma, and granulocytoma, were utilized. The search was implemented through the relevant scientific databases, including PubMed, UpToDate, and Scopus. As a result, 54 papers were specifically selected for inclusion in our manuscript, as they closely relate to our study. Articles written in the English language were included, while articles written in other languages were excluded.

## 3. Case 1

**Figure 1:**
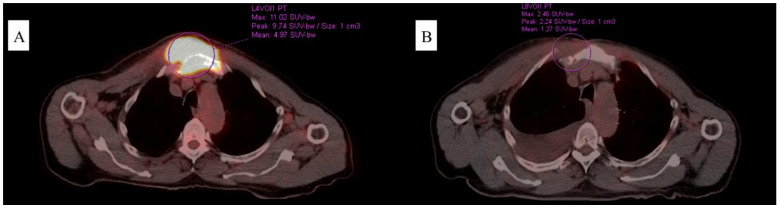
Comparison of the PET/CT scans upon diagnosis (**A**), and the best result achieved after treatment institution (**B**) related to Case 1.

**Figure 2:**
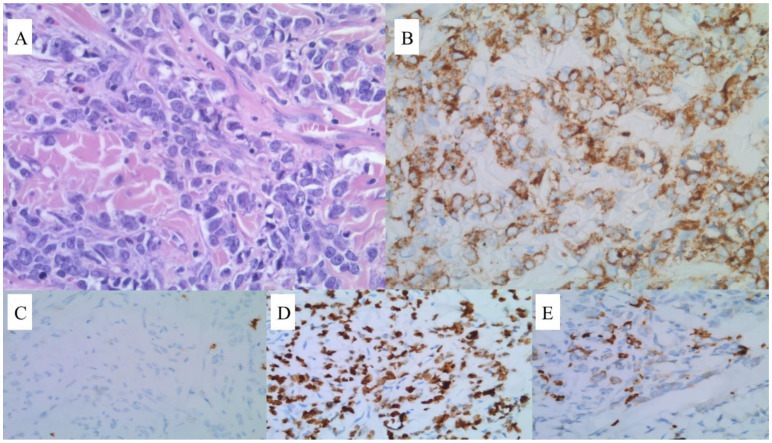
Microscopic examination of the tumor sample. (**A**) Diffuse proliferation of pleomorphic blasts with an irregular nuclear membrane. HE 400× magnification. (**B**) Strong CD34 expression. CD34 stain 400× magnification. (**C**) CD117-negative neoplastic cells. Staining is only visible in the reactive mast cells. (**D**) Weak expression of MPO in a subset of cells. (**E**) Ki-67 was positive in 80–90% of cells.

A 71-year-old man was admitted to our clinic to assess a growing mass in the area of the sternum, 6 months following their diagnosis of CML treated with imatinib in the standard dose of 400 mg/day (based on a medical interview, the patient had not brought documentation). A thorax computed tomography (CT) scan revealed a pathological mass located in the soft tissues on the right side of the chest. Furthermore, positron emission tomography/computed tomography (PET/CT) confirmed its presence, in addition to disclosing segmental lysis and the involvement of the manubrium of the sternum (Figure 1). Blood testing showed moderate normocytic anemia, lymphopenia, a low reticulocyte count, and high LDH and D-dimer levels (RBC 3.94 × 106/µL, HGB 11.2 g/dL, HCT 34.4%, MCV 87.3 fL, MCH 28.4 pg, LYM 0.58 × 103/µL, RET 0.22%, LDH 257 U/L, and D-dimer 730 ng/mL). In light of the clinical picture, a sample for pathology assessment was collected (Figure 2). Microscopic examinations revealed fibrous connective tissue covered by an infiltrate of cells with acidophilic cytoplasm and signs of angioinvasion. Immunohistochemical (IH) staining was positive for the following markers: CD45, CD34, CD117, CD15, MPO, Ki-67, and CK Pan, and was negative for CD3, CD20, PAX5, CD138, MUM1, S100, and CD30, respectively. BM biopsy excluded its involvement. Hence, a diagnosis of CML blast crisis in the form of MS was made. At the moment of MS diagnosis, BCR::ABL1 kinase domain sequencing revealed no mutations, which may have resulted in tyrosine kinase inhibitor (TKI) resistance. Upon receiving the results of RQ-PCR testing, which revealed b3a2 (p210) transcript levels of 5.891 (IS) corresponding to the absence of the major molecular response (MMR), the patient was subsequently deemed to be qualified for conformal radiotherapy (24 Gy in 12 fractions) in conjunction with dasatinib 140 mg/day.

**Figure 3:**
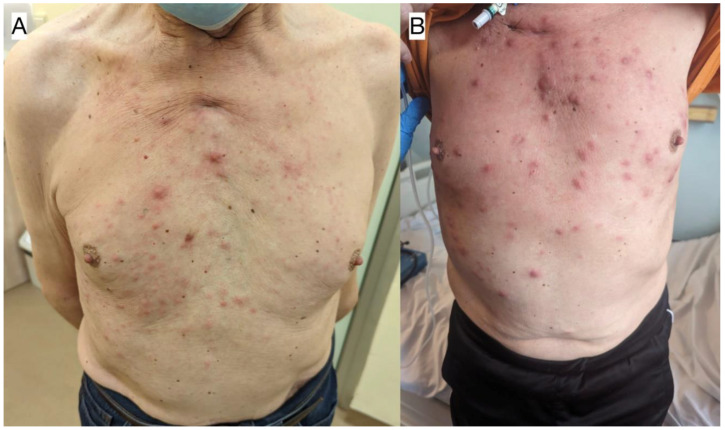
The evolution of the skin lesions observed in Case 1. (**A**) Skin lesions after developing resistance to dasatinib. (**B**) Progression of the skin lesions, shortly before the patient’s death.

After three months of dasatinib therapy, a major response to treatment was noticed both clinically, manifesting as a reduction in tumor volume, and molecularly, as p210 transcript levels dropped below the threshold of the deep molecular response (MR4). Nevertheless, the patient developed skin lesions (Figure 3A) and pleural effusion, which was confirmed with diagnostic imaging. Five months following therapy institution, BCR-ABL1 p210 transcripts reached a level of 1.45 (IS). Further scrutiny revealed the presence of the T315I (c.944C>T) mutation, thus explaining the cause of disease progression and the development of resistance to dasatinib. The patient was then qualified for being treated with ponatinib (45 mg/day), resulting in clinical and laboratory improvements, as one month into the new regimen, p210 transcript levels dropped, yet not below the MMR threshold. Due to a persistent presence of pleural effusion, flow cytometry upon sample collection was performed, showing the presence of blasts that were positive for CD15, CD65, CD13, CD34, CD117, HLA-DR, CD33, CD38, and CD7, and were negative for CD19 and MPO, respectively. Furthermore, 50% of the blast cells demonstrated altered signaling for the ABL1 and BCR genes, of which:

- Forty-three percent had four fusion signals, one red signal for ABL1 (9q34), and one green for BCR (22q11);
- Four percent had three fusion signals, one red signal for ABL1 (9q34), and one green for BCR (22q11);
- Three percent had two fusion signals, one red signal for ABL1 (9q34), and one green for BCR (22q11).

In the context of the cytogenetic testing performed upon diagnosis, these results are proof of clonal evolution and disease progression. Correspondingly, a regimen consisting of DA 3 + 7 (daunorubicin 60 mg/m^2^ days 1–3, Ara-C 100 mg/m^2^ days 1–7) and ponatinib 45 mg/day was administered, leading to the patient achieving the MR4 once more with real-time quantitative polymerase chain reaction (RQ-PCR) testing along with a concurrent resolution of dyspnea, which was present at the moment of admission.

**Figure 4:**
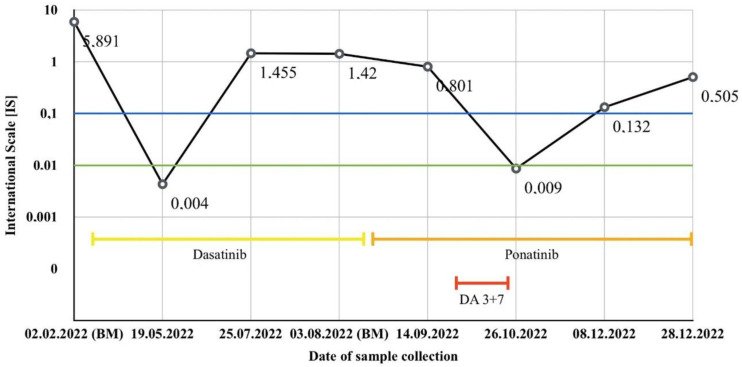
Results of BCR-ABL1 kinase domain sequencing related to Case 1. Samples were collected from the peripheral blood unless otherwise indicated. The blue line represents the MMR threshold, while the green one—MR4. Note: the patient has been administered dasatinib since 17 February 2022 (yellow line), ponatinib since 17 August 2022 (orange line), and CHTH following the DA 3 + 7 protocol between 8–14 October 2022 (red line).

Two months later, disease progression was revealed using RQ-PCR, CT, BM biopsy, and the evolution of skin lesions (as displayed in Figure 3B). Histopathological analysis of the skin lesions confirmed the relapse of MS in this patient. The reticular layer of the skin showed a diffuse infiltrate composed of CD34+ blastic cells showing weak expression levels of CD117 and CD15, accompanied with numerous neutrophil granulocytes. Blastic cell IH staining revealed: CD34 (+), CD 117 (+/−), CD15 (+/−), Ki-67 (+++)—reaction in 60–80% of the tumor cells, MPO (−), CD10 (−), CD79a (−), and CD3 (−). BCR::ABL1 kinase domain sequencing ruled out the presence of mutations, including the T315I mutation. Despite a satisfying response having been achieved following the reinduction of chemotherapy following the FLAG-IDA protocol (fludarabine 30 mg/m^2^, Ara-C 2 g/m^2^ for 5 days, idarubicin 10 mg/m^2^ for 3 days, and G-CSF 5 µg/kg days 1–5 and until neutrophil recovery), the patient unfortunately died, having subsequently suffered from neutropenia and respiratory tract infection leading to cardiorespiratory insufficiency. The results of BCR-ABL1 kinase domain sequencing have been presented in Figure 4.

## 4. Case 2

A 50-year-old male patient arrived at our hospital’s Hematology Department with a yet uncertain diagnosis, owing to an ambiguous result of histopathological analysis of a mediastinal tumor sample. The first and only symptom was reported to be noticed a little over 1 month prior to the admission to our ward, and it consisted of an enlargement of the neck’s circumference. A thorax CT scan was performed by a different clinic, revealing a mass located in the anterior mediastinum compressing the superior vena cava. Subsequently, the patient was referred to our hospital’s Department of Thoracic Surgery with symptoms of superior vena cava syndrome, which were managed with dexamethasone. Then, tumor sampling was performed, whose first analysis resulted being inconclusive, and the patient was subsequently transferred to our department.

**Figure 5:**
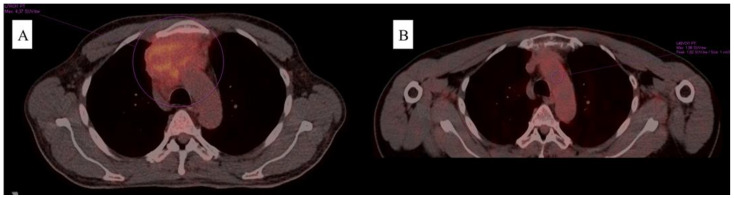
Comparison of the PET/CT scans upon diagnosis (**A**), and the best result achieved after treatment institution (**B**) related to Case 2.

**Figure 6:**
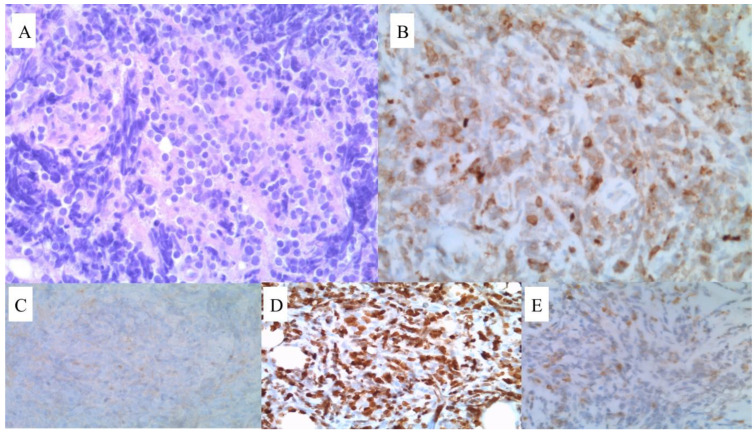
Microscopic examination of the tumor sample. (**A**) Diffuse infiltrate composed of monomorphic, medium-sized blasts. Note the strong crush artefact. HE 400× magnification. (**B**) Most cells weakly express CD79a. CD79a stain 400× magnification. (**C**) CD117-negative cells. (**D**) MPO stain was assessed as negative in most cells; however, some nonspecific stains were visible. (**E**) Ki-67 was positive in 80% of the cells.

Laboratory testing revealed moderate normocytic anemia with elevated LDH and D-dimer levels (RBC 4.24 × 10^6^/µL, HGB 13.4 g/dL, HCT 39.3%, MCV 92.7 fL, MCH 31.6 pg, LDH 277 U/L, and D-dimer 1040 ng/mL, respectively). As for the following diagnostic measures, we opted for PET/CT and BM biopsy (Figure 5). The former confirmed the presence of a mass in the anterior mediastinum with no signs of liver, spleen, BM, or lymph node involvement, while the latter revealed two blast populations, of which the first was positive for Tdt+, CD34, CD33, CD123, CD38, CD7, HLA-DR, and 45dim, and was negative for CD117, while the second one resulted positive for Tdt, CD34, CD33, CD123, CD38, CD7, and 45dim, and was negative for HLA-DR and CD117, respectively. Further scrutiny comprising G-banded karyotyping (GTG) of the BM found that the trisomy of chromosome 21 was present in 45% [9/20] of the metaphase cells. In the remaining 55% [11/20] of cells, a normal male karyotype was identified. Fluorescence in situ hybridization (FISH) revealed a triple signal for the RUNX1 gene (21q22) in 40% [51/127] of the interphase cells, indicating the presence of an extra copy of this gene. The evaluation of the signals retrieved from the metaphase cells confirmed the trisomy of chromosome 21. The presence of mutations, such as NPM1, BCR-ABL, FLT3-ITD, IDH 1/2, and the PML-RARa fusion gene, was excluded. Cerebrospinal fluid examination ruled out central nervous system involvement. A revision of the formerly collected tumor sample proved that the patient suffered from MS, and IH staining confirmed the cooccurrence of the same process both in the BM and in the anterior mediastinum (Figure 6). According to the clinical picture, a diagnosis of MS as the primary manifestation of a coexisting AML was made.

Due to an initial refusal of therapy, the patient was administered treatment following the DAC protocol (daunorubicin 60 mg/m2 days 1–3, cladribin 5 mg/m2 days 1–5, and Ara-C 200 mg/m2 days 1–7) one month after the diagnosis had been made. Despite a reduction in tumor volume and a lowering of blasts’ percentage in the BM, complete remission (CR) was not achieved. Therefore, we opted for a FLAG-IDA reinduction protocol (G-CSF 0.48 mg days 1–7, Ara-C 2 g/m2 days 2–6, fludarabine 30 mg/m2 days 2–6, and idarubicin 8 mg/m2 days 4–6) concomitantly with gemtuzumab ozogamicin (GO) (5 mg i.v. in day 1) resulting in a considerable expression of the CD33 antigen on the surface of the blast cells along with conformal radiotherapy (24 Gy in 12 fractions). This patient tolerated the regimen well, achieved CR, and was eligible for allogeneic peripheral blood stem cell transplantation (allo-PBSCT). Allo-PBSCT was delayed due to a gastrointestinal *Salmonella* spp. infection, which was resolved upon therapy within a few days. Transplantation from a matched unrelated donor with Bu4Cy2Thymoglobuline conditioning was performed eight months following the diagnosis with an unremarkable post-transplantation period.

Currently, at 8 months after allo-MUD-SCT, and 16 months since the diagnosis was made, the patient remains in CR with minimal residual changes in PET/CT imaging (Figure 3B), chimerism levels ranging 99.54–100%, and no viral reactivations observed.

## 5. Discussion

MS is frequently associated with CML or AML [1], and occasionally it can be their first manifestation, preceding the diagnosis of an ongoing myeloid malignancy [8]. In a clinical setting, it may be challenging to think of MS owing to its rarity and uncharacteristic clinical appearance. Histological examination and IH staining are two essential modalities, which in the case of MS usually reveal positive results for MPO, CD34, and c-kit. In both of our patients they had a fundamental role in making the diagnosis and ascertaining whether the extramedullary neoplasm corresponded histologically to the process in the BM.

Upon research through the scientific databases, we retrieved a total of 51 cases of MS in patients with either AML or CML considering the English-language literature, which we presented in Table 1 [9,10,11,12,13,14,15,16,17,18,19,20,21,22,23,24,25,26,27,28,29,30,31,32,33,34,35,36,37,38,39,40,41,42,43,44,45,46,47,48,49,50,51,52,53]. We sought to include recent articles that reported their outcomes with adequate clarity and in the light of the rapidly changing AML as well as CML treatment standards (including GO and 3rd generation TKIs). Of the fifty-one cases that were identified, seven of them concerned patients with CML, suggesting that MS occurs more frequently in the course of AML. The male-to-female ratio was 1:0.96. In terms of the time of MS onset, 18 cases were primary, 21 were secondary, and the remaining 12 were concomitant. Interestingly, every MS observed in the included CML cases was secondary, while in AML we noticed a prevalence of primary MS (n = 18) over secondary (n = 16) and cooccurring ones (n = 12). Several of the most commonly affected sites included the breast (n = 4), lymph nodes (n = 4), stomach (n = 3), and the retro-orbital space (n = 3). Bone involvement was observed in a total of six patients, and it included the temporal bone, maxilla, femur, thoracic spine, and twice the cranial base. With respect to therapy, CHTH was by far the most utilized approach (n = 31), in some instances was used in conjunction with surgical treatment (n = 7). It was also the most effective therapeutic approach, achieving CR in 15/31 cases. RT was employed in twelve patients, of whom nine received concomitant CHTH; its effectiveness was scarce, with CR achieved in only 3/12 cases. A less common, yet promising approach is allo-SCT, which was employed in 1/7 cases of MS with CML, and in 9/44 cases of MS and AML. CR was achieved in 5/10 cases including the one with CML, with an average follow-up period of 2.1 years ranging from 6 months to 5 years, respectively. An unusual method termed microtransplantation was instituted by Zhang et al. [22], comprising HLA-mismatched, G-CSF-mobilized, donor peripheral blood stem cell infusions following CHTH, with no graft-vs-host disease prophylaxis [54]. DP was observed in 19/51 cases, while PR was reported in 6/51 cases, respectively. However, 19/51 patients succumbed to their condition, with an average follow-up period of 0.56 year, ranging from 10 days to 3 years, respectively.

Considering the information retrieved from the found articles and our cases, we reached a few conclusions. Based on the available literature, it seems that patients with MS occurring in patients affected by AML are significantly more numerous than those with CML. Despite a claimed prevalence of MS in males over females with a ratio of 1.2:1 [6], we discovered it to be closer to 1:1, at least in the cases concerning coexisting AML or CML. An interesting finding we obtained was that considering the cited articles and our Case 1 patient, MS arose secondarily to CML in every instance, which strongly suggests that in the vast majority of patients with CML, MS will manifest secondarily rather than primarily. Regarding the Case 2 patient, the manifestation of MS briefly preceded the AML diagnosis, which is in line with the available data, where MS most frequently was primary to AML. As for the affected site, our cases present two extremely rare ones, as we found just one previous report of a patient suffering from mediastinal MS, and there have been none implicating MS of the sternum. Finally, it seems that the most frequently administered therapy was CHTH with concomitant RT in some instances along with surgical treatment if the mass results were particularly pronounced and symptomatic. An approach to be considered for the future is allo-SCT. Evidence coming from the analyzed studies and our Case 2 patient, who is currently in CR eight months after transplantation, suggests that allo-SCT can be seen as an effective, potentially curable treatment method. Interestingly, none of the included citations addressing MS-AML mentioned the inclusion of gemtuzumab therapy, which was administered in our Case 2 patient. Our results suggest that the addition of gemtuzumab may be an effective way to achieve and maintain CR in patients suffering from MS-AML. The inclusion of TKIs in MS-CML is reasonable but follows BCR::ABL1 kinase domain sequencing in search of mutations that could potentially rule out some TKI groups due to their inefficacy. Whenever the TKI-based regimen loses its efficaciousness, we have to take into account that a BCR-ABL mutation might have occurred in the course of the disease. Considering the MS-CML reports from Table 1, the T315I mutation was found in just 1/7 cases of MS-CML, and the inclusion of ponatinib therapy led to achieving clinical responses based on imaging. Unfortunately, our experience was different, as despite ponatinib treatment, patient 1 died due to septic complications following systemic chemotherapy. Taking into account both the rarity of MS-CML and the novelty of ponatinib therapy, our Case 1 report may be valuable in the context of opting for the appropriate therapy of MS-CML with the coexisting BCR-ABL1 mutation. It is important to note that MS can progress independently from the achievement of the molecular response, manifesting as a growth of the tumor.

We are aware that due to the very limited evidence available we cannot provide enough proof supporting a particular therapy or diagnostic pathway that could be considered the gold standard. Due to a scarce prevalence of MS, no randomized controlled trials can be conducted. Consequently, case reports are of major value to physicians, providing resourceful data on the clinical course of MS in the context of instituted therapeutic approaches. A particular focus should be put on molecular analysis and in reporting the outcomes of novel therapies, such as gemtuzumab in MS-AML and 3rd generation TKIs in instances of MS-CML with BCR-ABL1 kinase domain mutations. 

## Figures and Tables

**Table 1:** Case reports of patients affected by MS with myeloid malignancy.

Year	Author	Age	Sex	Malignancy	Occurrence of MS in Relation to Leukemia	Location	Follow-Up Period	Patient Status (Dead/Alive)	Therapy for MS	Clinical Outcome of MS
2022	Nanote et al. [9]	69	F	CML	Secondary	Parotid gland	NR	Alive	CHTH	NR
2022	Ali et al. [10]	26	M	CML, BP	Secondary	Maxilla	NR	Alive	CRT	PR
2020	Han et al. [11]	37	M	CML	Secondary	Spinal canal	1 year	Alive	Surgical, CHTH	CR
2019	Zhang et al. [12]	21	M	CML, T315I, BP	Secondary	Femur	NR	Alive	CHTH (ponatinib)	NR
2019	Palejwala et al. [13]	50	F	CML, BP	Secondary	Frontal lobe	1 month	Alive	Surgical, CRT	PR
2015	Ai et al. [14]	23	F	CML, BP	Secondary	Lymph nodes	4 years	Alive	CHTH, allo-SCT MRD	CR
2004	Kwatra et al. [15]	35	F	CML	Secondary	Breast	N/a	N/a	N/a	N/a
2022	Amiraian et al. [16]	63	F	AML	Secondary	Breast	NR	Dead	None (passed before therapy)	DP
2022	Mullen et al. [17]	26	F	AML	Primary	Uterine cervix	18 months	Alive	CHTH, allo-SCT MRD	CR
2022	Park et al. [18]	50	M	AML	Secondary	Conjuctiva	3 years	Alive	CRT, allo-SCT	CR
2022	Athukuri et al. [19]	30	M	AML	Secondary	Cranial base	8 months	Alive	Surgical, CHTH	PR
2022	Taminishi-Katsuragawa et al. [20]	66	M	AML	Concomitant	Stomach	NR	Alive	CHTH	CR
2022	Asawa et al. [21]	20	M	AML	Primary	Upper lobe of the left lung, Mediastinum	6 months	Alive	CHTH, allo-SCT	CR
2022	Zhang et al. [22]	38	F	AML	Primary	Lymph nodes	5.5 years	Alive	CHTH, microtransplantation [54]	DP
2022	Zhang et al. [22]	26	F	AML	Concomitant	Cranial base, parapharyngeal space	6 years	Alive	CHTH, microtransplantation [54]	CR
2022	Tuna et al. [23]	12	F	AML	Concomitant	Bladder	5 years	Alive	CRT, surgical, allo-SCT MUD	CR
2022	Ye et al. [24]	45	F	AML	Primary	Uterine cervix	1 year	Alive	Surgical, CHTH	CR
2022	Shash et al. [25]	4	M	AML	Primary	Retroperitoneum	4 years	Alive	CHTH	CR
2022	Wang et al. [26]	23	F	AML	Concomitant	Liver	NR	Dead	CHTH	CR
2021	Cross et al. [27]	45	M	AML	Primary	Heart, Pancreas	NR	Alive	CHTH	PR
2021	Gosavi et al. [28]	27	M	AML	Secondary	Prostate	6 months	Dead	CRT, allo-SCT	DP
2021	Huang et al. [29]	34	F	AML	Secondary	Breast	1 year	Dead	CHTH, allo-SCT MMRD	DP
2021	Wu et al. [30]	32	F	AML	Secondary	Pancreas	NR	Alive	CHTH	PR
2021	Thomson et al. [31]	67	M	AML	Primary	Testicles	NR	Alive	CHTH	CR
2020	Liu et al. [32]	15 months	M	AML	Primary	Nasal cavity	2 years	Alive	Surgical, CHTH	CR
2020	Hernández et al. [33]	22	F	AML	Concomitant	Intestine	12 days	Dead	Surgical	DP
2020	Sun et al. [34]	37	M	AML	Secondary	Retro-orbital space	6 months	Dead	RT, allo-SCT	DP
2020	Slusarenko da Silva et al. [35]	29	M	AML	Primary	Temporal region	N/a	N/a	N/a	N/a
2020	Kumar et al. [36]	64	F	AML	Secondary	Thoracic spine	NR	Dead	CRT	DP
2020	Pi et al. [37]	30	F	AML	Concomitant	Lymph node	4 months	Dead	CHTH	DP
2019	Agarwal et al. [38]	72	F	AML	Primary	Bile ducts	2 months	Dead	Surgical	DP
2019	Marwah et al. [39]	2	M	AML	Primary	Temporal bone	NR	Alive	CHTH	CR
2019	Almalki et al. [40]	1	M	AML	Concomitant	Retro-orbital space	NR	Alive	CHTH	N/a
2019	Abdelnabi et al. [41]	70	M	AML	Secondary	Left cardiac ventricle	NR	Dead	CRT	DP
2019	Bubulac et al. [42]	30	F	AML	Secondary	Breast	3 years	Dead	CRT, allo-SCT MRD	DP
2019	Feng et al. [43]	56	M	AML	Secondary	Lung	N/a	N/a	CHTH	CR
2018	Nguyen et al. [44]	73	M	AML	Primary	Prostate	NR	Alive	Surgical, CHTH	PR
2018	Khaja et al. [45]	29	F	AML	Concomitant	Lymph node	NR	Alive	CHTH	NR
2017	Siraj et al. [46]	38	M	AML	Primary	Nasal cavity	NR	N/a	CHTH	N/a
2017	Siraj et al. [46]	11	M	AML	Primary	Retro-orbital space	18 months	Alive	CRT	CR
2017	Siraj et al. [46]	49	M	AML	Primary	Brain	6 months	Dead	Surgical, CHTH	DP
2017	Siraj et al. [46]	52	M	AML	Secondary	Peritoneum	NR	NR	NR	NR
2014	Huang et al. [47]	58	F	AML	Primary	Duodenum	NR	Alive	CHTH	CR
2012	Kini et al. [48]	25	M	AML	Concomitant	Gastrointestinal tract	10 days	Dead	Surgical	DP
2011	Cash et al. [49]	24	F	AML	Secondary	Heart	NR	Dead	CHTH, allo-SCT	DP
2011	Gadage et al. [50]	35	M	AML	Concomitant	Stomach	10 months	Dead	CHTH	DP
2011	Gadage et al. [50]	25	M	AML	Primary	Stomach	15 days	Dead	Surgical	DP
2010	Zhang et al. [51]	16	M	AML	Concomitant	Abdomen	NR	Alive	CHTH	CR
2010	Skeete et al. [52]	77	F	AML	Primary	Vagina	5 months	Dead	RT	DP
2010	Skeete et al. [52]	36	F	AML	Secondary	Vagina	5 months	Dead	RT	DP
2009	Mneimneh et al. [53]	65	M	AML	Concomitant	Prostate	3 weeks	Dead	Surgical, CHTH	DP

Abbreviations: CHTH—chemotherapy, CRT—chemoradiotherapy, RT—radiotherapy, allo-SCT—allogeneic stem cell transplantation, MUD—matched unrelated donor, MRD—matched related donor, MMRD—mismatched related donor, DP—disease progression, CR—complete remission, PR—partial response, and BP—blast phase upon MS diagnosis, T315I—T315I mutation (in CML), N/a—not available, and NR—not reported.

## Data Availability

Not applicable.
